# A LONGITUDINAL ANALYSIS OF DOMAIN-SPECIFIC CHANGES IN PROMIS® SELF-EFFICACY AND PREDICTORS IN PARKINSON’S DISEASE

**DOI:** 10.1093/geroni/igae098.3776

**Published:** 2024-12-31

**Authors:** Haesung Kim, Lisa Shulman, Laurence Magder, John Schumacher, George Unick, Sunita Shakya, Ann Gruber-Baldini

**Affiliations:** University of Maryland Baltimore, Baltimore, Maryland, United States; University of Maryland School of Medicine, Baltimore, Maryland, United States; University of Maryland Baltimore, Baltimore, Maryland, United States; University of Maryland Baltimore County, Baltimore, Maryland, United States; University of Maryland Baltimore, Baltimore, Maryland, United States; University of Maryland Baltimore, Baltimore, Maryland, United States; University of Maryland School of Medicine, Baltimore, Maryland, United States

## Abstract

**Background:**

There is limited research on the magnitude and predictors of longitudinal changes in Self-Efficacy (SE) among Parkinson’s disease (PD) patients. OBJECTIVE: To describe longitudinal changes in Patient-Related Outcomes Measurement Information System -SE (PROMIS-SE®): managing daily activities (SEM-DA), emotions (SEM-E), medications and treatments (SEM-MT), social interactions (SEM-SO), and symptoms (SEM-S) and examine predictors of changes in PD.

**Methods:**

A longitudinal analysis was conducted on 239 PD patients using two-time points (3years±180days). Baseline predictors included age, sex, race, education, PD duration, PD severity, medical comorbidities, and cognitive ability. Student T-tests and Pearson’s correlation coefficient examined the relationship between PROMIS-SE domains and predictors of changes.

**Results:**

SE decreased by more than 5 T-score units (0.5SD) across all domains [Mean±SD: SEM-DA (-6.13±8.66), SEM-E (-5.32±10.49), SEM-MT (-7.2±10.11), SEM-SO (-5.69±9.96), and SEM-S (-5.18±10.25)]. Significant larger mean declines (p<.05) were associated among older age (age< 65 vs. 65+) in SEM-E (-3.84 vs. -6.53) and SEM-MT (-5.63 vs. -8.47), higher cognition (MoCA< 26 vs.26+) in SEM-E (-1.54 vs. -7.30), SEM-SO (-3.22 vs. -7.03), and SEM-S (-3.17 vs. -6.58), and higher education (no college vs. college) in SEM-SO (-2.10 vs. -6.00). Change in UPDRS total score correlated with change in SEM-S (r=-0.26, p<.05).

**Conclusions:**

Over three years, PD patients experienced significant SE changes across all domains, with the greatest decline in SE for managing medical treatments. Greater declines in SE were associated with older age, higher cognition, and higher education. This may indicate a higher sensitivity of the SE measure to change among those with higher cognition and education.

